# Unveiling potential: urinary exosomal mRNAs as non-invasive biomarkers for early prostate cancer diagnosis

**DOI:** 10.1186/s12894-024-01540-6

**Published:** 2024-08-01

**Authors:** Jiayin Yu, Chifei Yu, Kangxian Jiang, Guanglin Yang, Shubo Yang, Shuting Tan, Tingting Li, Haiqi Liang, Qihuan He, Faye Wei, Yujian Li, Jiwen Cheng, Fubo Wang

**Affiliations:** 1 Department of Urology, The First Affiliated Hospital of Guangxi Medical University, No.6 Shuangyong Road, Qingxiu, Nanning, Guangxi 530021 P.R. China; 2 Department of Urology, Affiliated Tumor Hospital of Guangxi Medical University, No.71 Hedi Road, Qingxiu District, Nanning, Guangxi 530021 P.R. China; 3 Department of Urology, The Second Affiliated Hospital of Fujian Medical University, No. 34 Zhongshan North Road, Quanzhou, Fujian 362000 P.R. China; 4 Center for Genomic and Personalized Medicine, Guangxi Key Laboratory for Genomic and Personalized Medicine, Guangxi Medical University, No.22 Shuangyong Road, Qingxiu District, Nanning, Guangxi 530021 P.R. China

**Keywords:** Prostate, Cancer, Biomarker, mRNA, Urine, Exosome

## Abstract

**Background:**

This study investigated the use of urinary exosomal mRNA as a potential biomarker for the early detection of prostate cancer (PCa).

**Methods:**

Next-generation sequencing was utilized to analyze exosomal RNA from 10 individuals with confirmed PCa and 10 individuals without cancer. Subsequent validation through qRT-PCR in a larger sample of 43 PCa patients and 92 healthy controls revealed distinct mRNA signatures associated with PCa.

**Results:**

Notably, mRNAs for *RAB5B*, *WWP1*, *HIST2H2BF*, *ZFY*, *MARK2*, *PASK*, *RBM10*, and *NRSN2* showed promise as diagnostic markers, with AUC values between 0.799 and 0.906 and significance p values. Combining *RAB5B* and *WWP1* in an exoRNA diagnostic model outperformed traditional PSA tests, achieving an AUC of 0.923, 81.4% sensitivity, and 89.1% specificity.

**Conclusions:**

These findings highlight the potential of urinary exosomal mRNA profiling, particularly focusing on *RAB5B* and *WWP1*, as a valuable strategy for improving the early detection of PCa.

**Supplementary Information:**

The online version contains supplementary material available at 10.1186/s12894-024-01540-6.

## Introduction

Prostate cancer (PCa) is a significant global health concern, affecting millions of men worldwide with over one million new cases diagnosed annually. It is a serious and life-threatening disease, ranking as the second leading cause of death among men [1, 2]. The challenges of tumor progression and drug resistance necessitate early detection and treatment to enhance patient survival and quality of life [3, 4]. While the PSA test is frequently utilized, it is important to acknowledge the limitations of relying solely on a PSA level of 4–20 ng/ml and prostate biopsy. This approach can lead to both overdiagnosis and underdiagnosis of prostate cancer. [5, 6]. Consequently, researchers have dedicated efforts toward identifying noninvasive liquid biopsy markers for prostate cancer to enhance early diagnosis and treatment efficacy. This approach not only circumvents the discomfort associated with biopsy procedures but also provides a more comprehensive understanding of tumor heterogeneity [7, 8].

The use of exosome markers in noninvasive liquid biopsy techniques has garnered significant attention in recent research. Exosomes, small vesicles measuring between 30 and 150 nm in diameter, are released by cells and carry a variety of biomolecules including proteins, lipids, and nucleic acids. Encased in a lipid bilayer membrane, these vesicles protect their cargo from degradation by nucleases, playing a crucial role in intercellular communication [9, 10]. The exceptional stability of exosomes has also led to their exploration as delivery vehicles for targeted tumor therapy [11, 12]. Studies have highlighted the diagnostic and prognostic potential of exosomal long non-coding RNAs (lncRNAs) *MALAT1* and *HOTAIR* in serum exosomes, offering valuable insights for tumor assessment via liquid biopsies [10]. These lncRNAs have been implicated in regulating various biological processes in prostate cancer through interactions with miRNAs, proteins, and other molecules [13] Additionally, the regulatory role of exosomal *HOXD-AS1*-mediated *miR-361-5p/FOXM1* in prostate cancer has been elucidated [14]. Furthermore, *miR-1290* and *miR-375* in exosomes hold promise as prognostic markers for prostate cancer survival [15], while exosomal overexpression of PD-L1 has been associated with cancer progression in multiple malignancies [16, 17]. Notably, the presence of exosomes in urine was initially discovered by Pisitkun et al., who identified exosomes as convenient and accessible sources for urine exosome sample collection and processing. This breakthrough has opened up new possibilities for early cancer detection [18]. Subsequently, Lee GL et al. confirmed the diagnostic potential of *PCA3* as a marker for prostate cancer progression [19]. Donovan MJ and colleagues, including Sanda MG, demonstrated that identifying exosomal *PCA3* in urine and assessing *T2:ERG* expression levels in urine can aid in the early detection of prostate cancer, as well as monitoring disease progression and recurrence [19–21]. However, the exploration of urinary exosomal mRNA markers in prostate cancer patients remains relatively limited.

Urinary exosomal mRNA holds great promise as a marker for prostate cancer, offering several advantages. Firstly, urine collection is a simple and convenient process, making it suitable for mass screening and early detection efforts. Secondly, exosomal mRNA exhibits remarkable stability and shows resistance to degradation. The study proposes collecting urine samples from prostate cancer patients after either a prostate massage or digital rectal examination (DRE). This study aimed to enhance the accuracy of tumor status assessment and expedite the detection of prostate cancer by analyzing exosomal mRNA expression levels in urine samples. The objective of this study was to establish a comprehensive diagnostic protocol for post-prostate massage urinary exosomal mRNA, which could revolutionize early detection and treatment of prostate cancer through further research and medical validation. This framework introduces an innovative approach and technique in the field.

## Methods

### Clinical samples and urine collection, cell lines

**Fig. 1:**
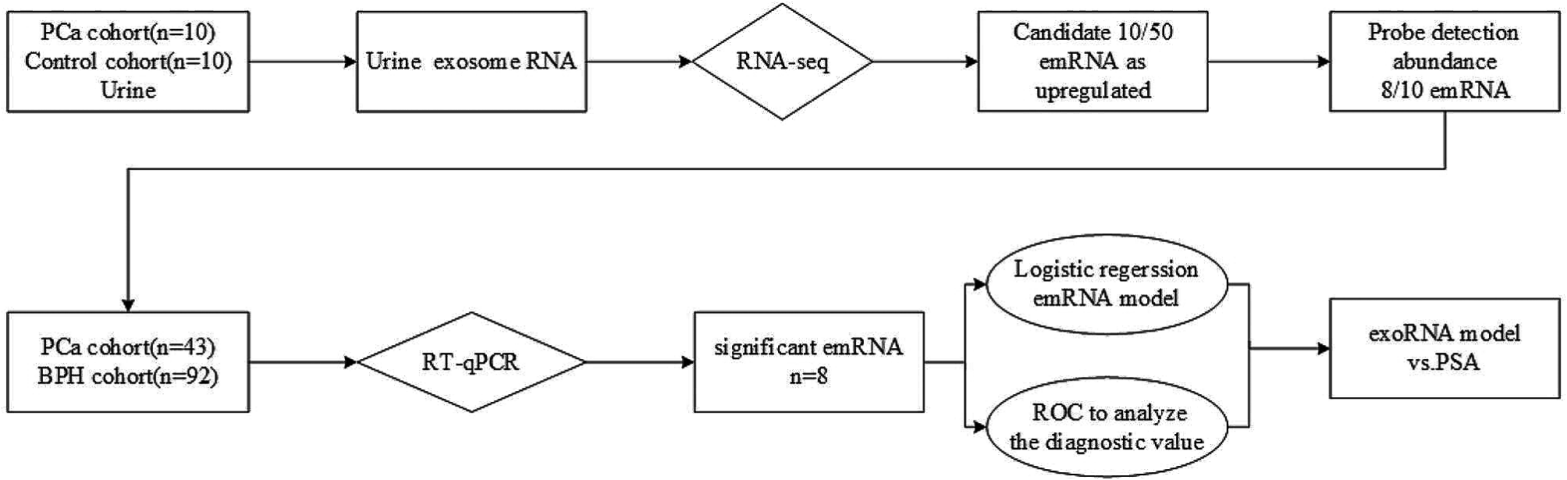
Workflow

Before sample collection, the research was ethically reviewed by the Institutional Committee for Human Research Ethics at the First Affiliated Hospital of Guangxi Medical University and the Cancer Hospital affiliated with Guangxi Medical University. All sites followed identical procedures, as per the standard operating manual (SOP), for recruiting participants, processing samples, and conducting prostate biopsies. Participants eligible for biopsies were those with a PSA increase of over 4 ng/mL or those with a normal PSA level but suggestive DRE findings of a nodule or abnormal imaging results. Prostate cancer (PCa) and benign prostate hyperplasia (BPH) samples were verified through biopsies, and two pathologists analyzed the biopsy tissues to confirm the diagnosis and determine the Gleason score.All subjects provided informed consent, and the sample collection and research procedures (Fig. 1) followed authorized clinical protocols.

Urine samples were collected from 110 individuals at the First Affiliated Hospital of Guangxi Medical University and 45 patients at the Tumor Hospital of Guangxi Medical University between June 2016 and June 2019. Initially, 40 milliliters of urine were stored at cold temperatures, followed by centrifugation at 4 °C and 2500 g for 10 min. The resulting supernatant was then frozen at -80 °C for future analysis. Prostate cancer cell lines C4-2B and PC-3, as well as normal prostate epithelial cells RWPE-1, were obtained from the Cell Bank of the Chinese Academy of Medical Sciences in Shanghai. RWPE-1 cells were cultured in keratinocyte-serum-free medium (K-SFM; Gibco), while C4-2B and PC-3 cells were maintained in RPMI-1640 medium (Gibco, Carlsbad, CA, USA). All cell lines were grown in a solution containing 10% fetal bovine serum (FBS; Gibco), 100 U/mL of penicillin, and 100 µg/mL of streptomycin (Invitrogen) at 37 °C with 95% air and 5% CO2.

### Isolation of exosomes from urine supernatants

Ultracentrifugation is the most commonly used method to extract exosomes from urine [22]. The frozen urine sample was initially thawed at -80 °C, then cooled to 4 °C before being centrifuged at 300 g for 10 min. Subsequent centrifugations were conducted at 2500 g for 10 min, 10,000 g for 30 min, and 100,000 g for 90 min using ultracentrifuge settings. The resulting supernatant was collected, and the exosomes were re-suspended in 1× PBS, adjusted to the appropriate volume, and passed through a 0.22 μm filter (Millipore, SLGVR33RB) into an ultracentrifuge tube. Another round of ultracentrifugation at 100,000 g for 70 min was performed, followed by resuspension in 250 µl of PBS, gentle mixing, and storage at -80 °C for future use.

### Transmission electron microscopy (TEM)

Subsequently, the urinary system was examined using a JEOL JEM-1010 transmission electron microscope (JEOL USA INC., Peabody, MA) after air-drying at room temperature following the application of 20 µL of a 3% solution of phosphotungstic acid or 10 µL of a 2% aqueous solution of hydrogen peroxide and acetic acid at ambient room conditions for one minute. Excess liquid was removed from the surface using filter paper.

### Nanoparticle-tracking analysis(NTA)

Exosomes were suspended in Phosphate buffer solution (PBS) and filtered through a syringe filter (Millipore) before being further concentrated to visualize individual nanoparticles. The size distribution of the urinary exosomes was analyzed using the NanoSightNS300 instrument from Malvern Instruments Ltd. in Worcestershire, UK.

### Western blot (WB)

To identify specific exosomal markers, antibodies targeting CD63 and TSG101 were obtained from Abcam in Cambridge, UK. Urinary exosomes were lysed using a cold lysis buffer (Beyotime; P0013), followed by mixing protein samples with a loading buffer (Tanon; 180-8210D). The subsequent steps included separation through SDS-PAGE and transfer onto a PVDF membrane via an electric process. The membranes were blocked with 5% skimmed milk and then incubated overnight with a primary immunoblotting antibody at 4 °C, followed by exposure to an HRP-conjugated secondary antibody from Cell Signaling Technology in the USA. Visualization of the blots was achieved using the Chemistar Visualize™ High Signal ECL Protein Blotting Substrate Kit (Tanon; 180–501). The Western Blot bands in this study were edited primarily because of protein degradation that occurred during the experimental process, the use of non-fluorescent pre-stained protein markers in the experiment, and the aim to reduce costs.

### Next-generation urinary exosome RNA sequencing

RNA was extracted from the urinary exosomes using a urinary exosome RNA isolation kit (Norgen Biotek Corp. #47,200), according to the manufacturer’s instructions. The Illumina NEBNext UltraII RNA library preparation kit (NEB, #E7770S) was then used for library construction. Total RNA sequencing was performed by the ExoNGS service offered by SystemBiosciencesInc. Data analysis was conducted using the Maverix Analysis Platform, a cloud-based RNA-SEQ analysis kit from System Biosciences Inc.

### RNA extraction and quantitative real-time PCR (qRT-PCR)

Cell lysis was conducted using Trizol^®^ reagent (Life Technologies, Australia) for total RNA extraction, with the addition of chloroform to facilitate RNA separation. The RNA was then dissolved in 50 µL of RNase-free water post-precipitation. The quality and integrity of the RNA were evaluated using a NanoDrop spectrophotometer (Thermo Fisher Scientific, Australia). For cDNA synthesis, 1 µg of total RNA was reverse transcribed with the PrimeScript RT kit (Takara Holdings, Kyoto, Japan). qRT-PCR analysis was carried out using 2× TaqMan-qPCR Master Mix (Solarbio) as per the provided instructions, with cycling parameters on a CFX96 touch instrument. Ct values exceeding 38 were disregarded, with *ACTB* serving as the internal control gene. The 2-ΔΔCt method was employed to determine the expression ratio of the target gene. Primers were supplied by Sangon Bioengineering Co. (Shanghai, China), and PCR products were assessed through gel electrophoresis on 2% agarose gels after the experiment was replicated three times.

### Statistical analysis

Utilizing SPSS Statistics 26.0, GraphPad Prism 8.0, and R (v. 4.2.3), the research team conducted a comprehensive analysis of the data. Various statistical tests were performed to compare data sets and correlation coefficients were calculated to explore relationships between variables. Diagnostic accuracy was assessed through both one-way and multifactorial logistic regression analyses. The team also examined the characteristics of the subjects’ work using ROC curve analysis to determine the AUC. Significance was set at p-value < 0.05 for all findings.

## Results

### Urinary exosomal characteristics

**Fig. 2:**
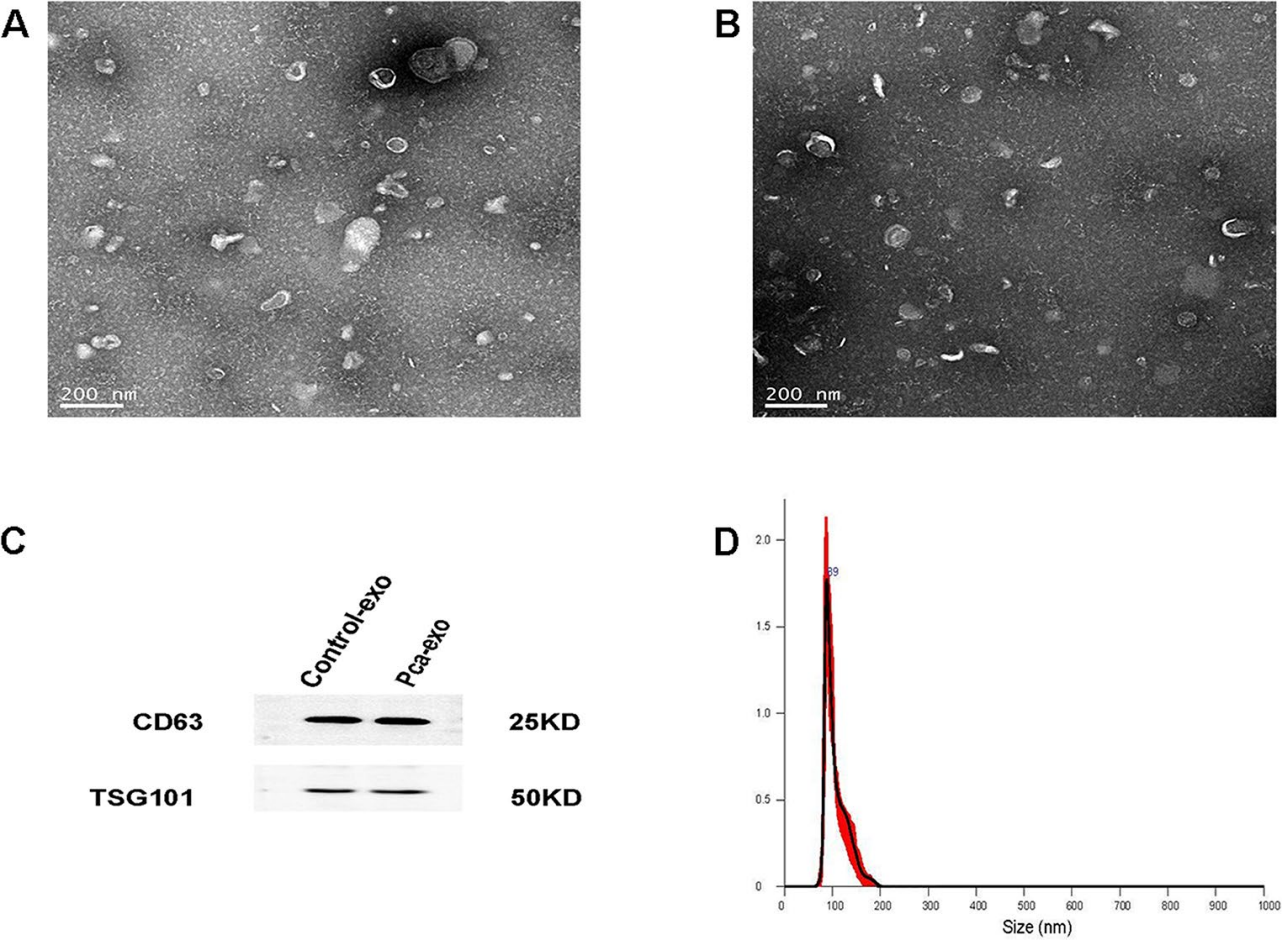
Identification of exosomes in urine. (**A**,**B**) TEM image of samples from healthy individuals revealing the presence of exosomes and PCA. (**C**) Exosomes obtained from healthy participants and PCA patients express CD63 and TSG101 proteins, as indicated by Western blot analysis. (**D**) Assessment of exosome levels in urine using NTA

TEM analysis revealed that exosomes exhibited a cup-shaped vesicular morphology, measuring approximately 100 nm in diameter. The vesicles displayed a distinct double-layered membrane structure and were found to be stably present in the PBS suspension (Fig. 2A-B). The presence of exosome markers, CD63 and TSG101, was confirmed through protein blotting analysis of the exosome samples (Fig. 2C) [23, 24]. Furthermore, NTA demonstrated that these exosomes accounted for over 90% of the exosomes within the size range of 30–150 nm. This finding aligns with the previously reported size distribution of exosomes (Fig. 2D) [25, 26]. Collectively, these results provide compelling evidence for the existence of exosomes in urine and lay the groundwork for future investigations into exosome biomarkers.

### Screening and analysis of mRNAs of urinary exo origin

**Fig. 3:**
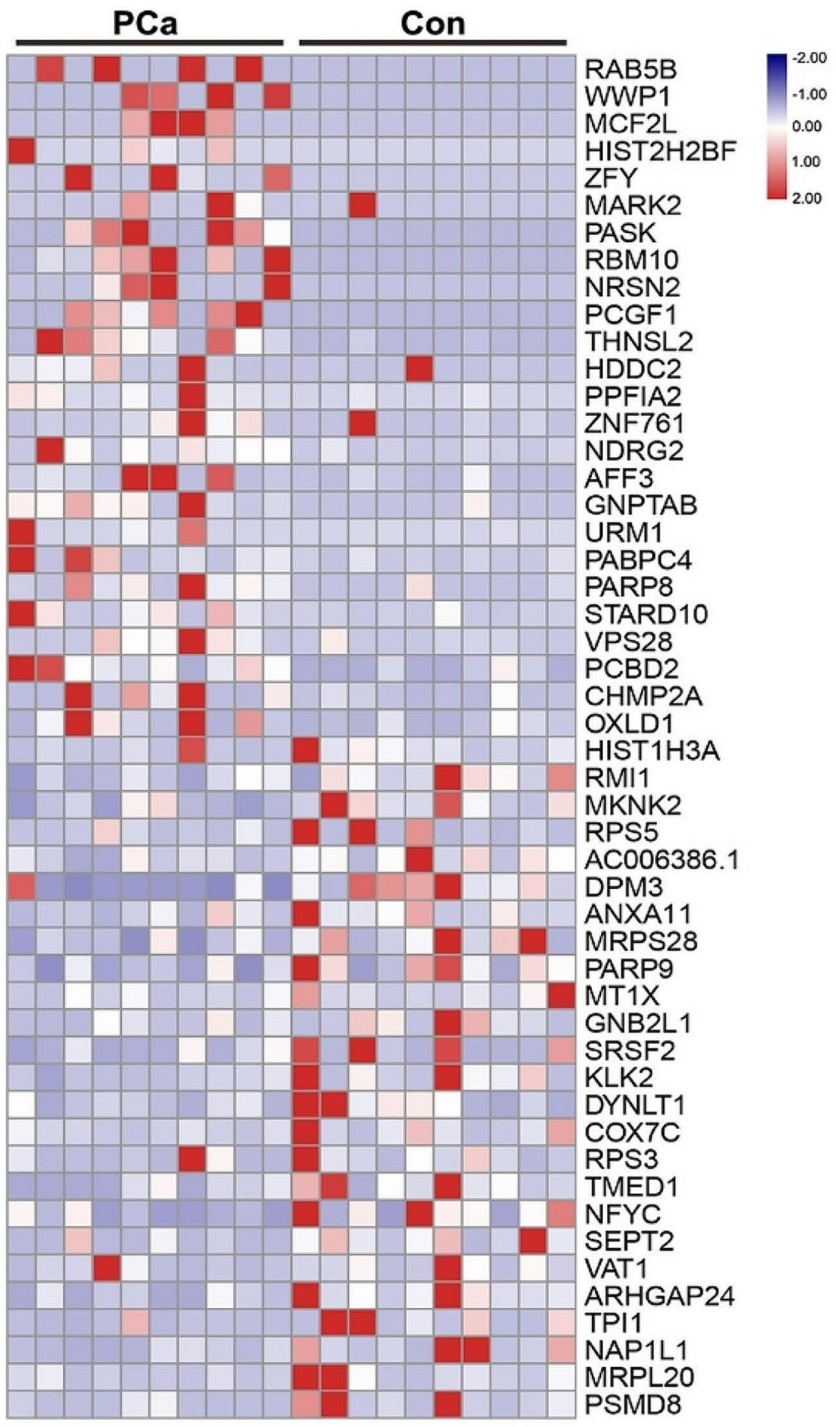
Urinary exosomes mRNAs heatmap of the microarray

In this study, we aimed to identify differential mRNAs by analyzing the urinary exosomal transcriptomes of 10 prostate puncture-positive and 10 puncture-negative patients using second-generation sequencing. Based on their expression levels, a total of 429 mRNAs were found to be up-regulated, while 498 mRNAs were down-regulated. To visualize the expression patterns of the top 50 significantly up-regulated mRNAs, a heatmap was generated (Fig. 3). Furthermore, the top 10 mRNAs exhibiting the most significant differences associated with prostate cancer were selected as candidate genes. These genes included *RAB5B*, *WWP1*, *MCF2L*, *HIST2H2BF*, *ZFY*, *MARK2*, *PASK*, *RBM10*, *NRSN2*, and *PCGF1*. To ensure the targeted and stable detection of the screened mRNAs, we optimized the RNA assay methodology. Firstly, we analyzed the abundance distribution of these 10 candidate genes in urinary exosomal RNA and visualized each exon of the genes using Integrative Genomics Viewer (IGV) (Supplemental Material 1). Specific primers and probes (Supplemental Material 2) were then designed for qRT-PCR detection, aiming to obtain exosomal mRNA fragments with the highest expression abundance and a single band (Supplemental Material 3). Subsequently, we employed qRT-PCR to validate the presence of these genes in patient urinary exosomes, exosomes from PCa cell lines (C4-2B and PC-3), and exosomes from RWPE-1, a normal prostate cell line. The results indicated that only eight genes, namely *RAB5B*, *WWP1*, *HIST2H2BF*, *ZFY*, *MARK2*, *PASK*, *RBM10*, and *NRSN2*, were detectable as relevant amplification products. To further confirm the accuracy of these findings, we used exosomal cDNA from the RWPE-1 cell line to detect valid probes for these eight genes and performed DNA gel electrophoresis of the qRT-PCR products. The electrophoresis results were consistent with the detected amplification products of the target genes.

### Validation of mRNA gene expression levels in urinary exosomes

**Fig. 4:**
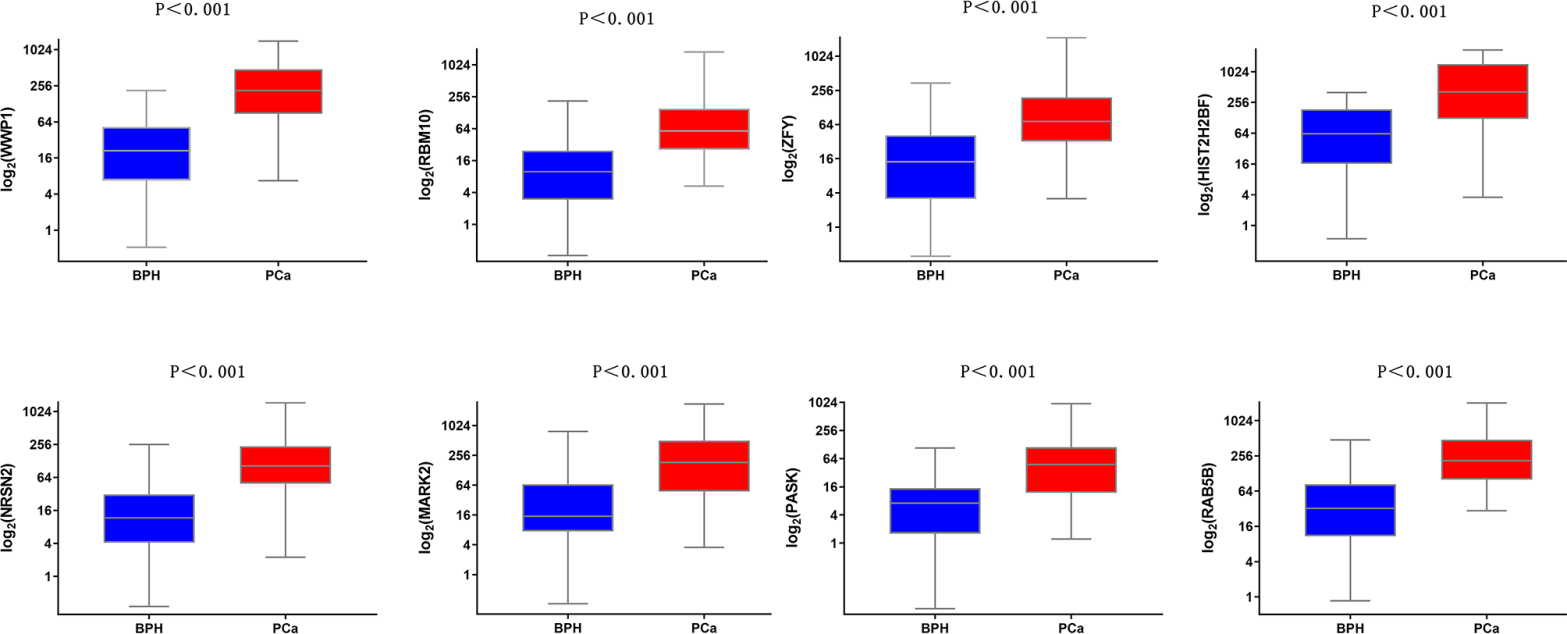
The mRNA expression levels in exosomes from the urinary tract were significantly higher in the prostatic hyperplasia group (*p <* 0.001). The mRNAs include *WWP1*,* RBM10*,* ZFY*,* HIST2H2BF*,* NRSN2*,* MARK2*,* PASK*, and *RAB5B*

Next, the expression of these 8 mRNAs in 43 PCa patients and 92 healthy controls was evaluated by qRT-PCR in this study. Statistical analysis showed that the puncture positivity rate was 31.9% in 135 samples. *WWP1*, *RBM10*, *ZFY*, *HIST2H2BF*, *NRSN2*, *MARK2*, *PASK*, and *RAB5B* were relatively highly expressed in the PCa group compared to the prostate hyperplasia group and were statistically significant in terms of difference (*P <* 0.001)(Supplemental Material 5 and Fig. 4). These eight mRNAs (*WWP1*, *RBM10*, *ZFY*, *NRSN2*, *MARK2*, *PASK*, *HIST2H2BF*, and *RAB5B*) were searched in literature databases, and were found to play a functional role in tumorigenesis [27–34]. Taken together, these findings suggest that the screened mRNAs are of some significance, but whether they can be used as PCa-independent risk factors is unclear.

### Validation of the diagnostic efficacy of urinary exosomal mRNA in PCa patients

To determine whether these eight mRNAs could serve as independent risk factors for PCa, we conducted a one-way logistic regression analysis. The results of the analysis demonstrated that *WWP1*, *RBM10*, and *ZFY* (*P <* 0.001) among the eight candidate molecules could function as independent predictors of PCa (Table 1).

**Fig. 5:**
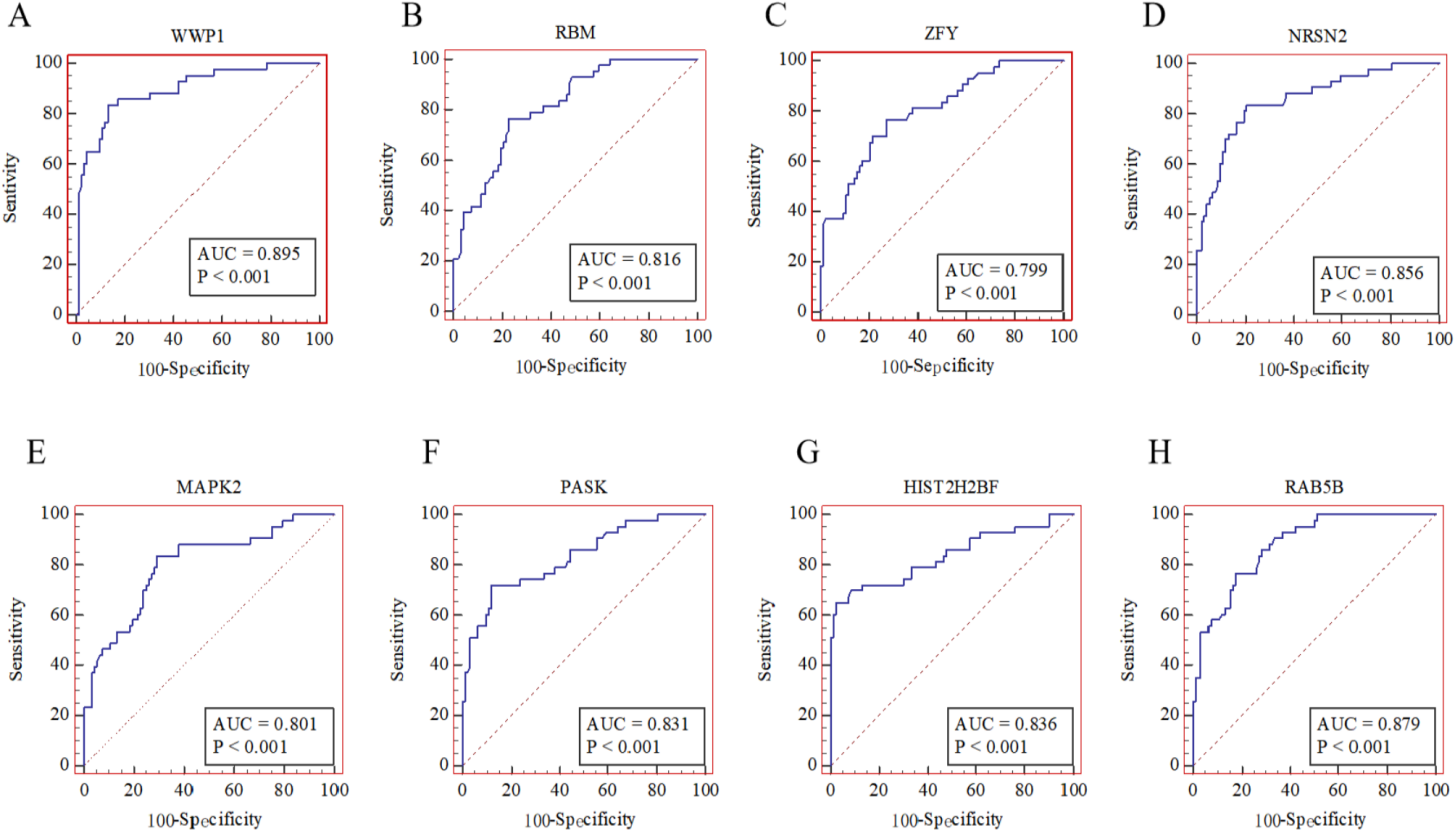
Using ROC-AUC to analyze the diagnostic value of independent predictors in PCa

To evaluate the diagnostic potential of mRNA markers for PCa, ROC analysis was performed. The results (Fig. 5) revealed that *WWP1* had the highest AUC value of 0.906 (95% CI = 0.850–0.963, 83.72% sensitivity, and 88.04% specificity) among the eight genes. Following closely was *RAB5B* with an AUC of 0.880 (95% CI = 0.823–0.936, sensitivity 76.74%, specificity 82.61%), while *ZFY* had the lowest AUC of 0.799 (95% CI = 0.719–0.87, sensitivity 76.74%, specificity 72.83%). Among the genes, *NRSN2* showed the highest sensitivity of 83.72% (AUC = 0.856, 95% CI = 0.786–0.925, specificity 79.35%), whereas *HIST2H2BF* exhibited the highest specificity of 97.83% (AUC = 0.835, 95% CI = 0.752–0.919, sensitivity 65.12%). Conversely, *MARK2* had the lowest specificity at 70.65% (AUC of 0.801, 95% CI: 0.720–0.883, sensitivity 83.72%). *PASK* had an AUC of 0.831 (95% CI = 0.755–0.908, sensitivity 72.9%, specificity 88.04%), RBM showed an AUC of 0.817 (95% CI: 0.745–0.889, sensitivity 76.74%, specificity 77.17%), and PSA had an AUC of 0.606 (95% CI: 0.498–0.715, sensitivity 62.79%, specificity 64.13%). It is noteworthy that the lower AUC value of PSA aligns with its reported range in the literature as a PCa biomarker (AUC range from 0.54 to 0.70) [35]. Overall, the diagnostic performance of the eight potential mRNA markers for urinary exosomes exceeded that of PSA (AUC = 0.606, 95% CI: 0.498–0.715, sensitivity 62.79%, specificity 64.13%).

**Table 1:** Univariate and multivariate logistic regression analysis and AUC of urinary exosome mRNAs

Index group	Univariate				Multivariate
	*P*	AUC	Sensitivity (%)	Specificity (%)	*P*
PSA	0.033	0.606	62.79	64.13	0.106
*WWP1*	<0.001	0.906	83.72	88.04	0.001
RBM	<0.001	0.817	76.74	77.17	0.983
*ZFY*	<0.001	0.799	76.74	72.83	0.164
*NRSN2*	<0.001	0.856	83.72	79.35	0.225
MARK2	<0.001	0.801	83.72	70.65	0.203
*PASK*	<0.001	0.831	72.09	88.04	0.659
*HIST2H2BF*	<0.001	0.835	65.12	97.83	0.89
*RAB5B*	<0.001	0.880	76.74	82.61	0.025

PSA: Prostate-specific antigen; OR : Odds ratio ;95% CI :95% Confidence interval; AUC :Area under the receiver operating characteristic curve

### Establishment and validation of a diagnostic model based on urinary exosomal mRNAs

**Fig. 6:**
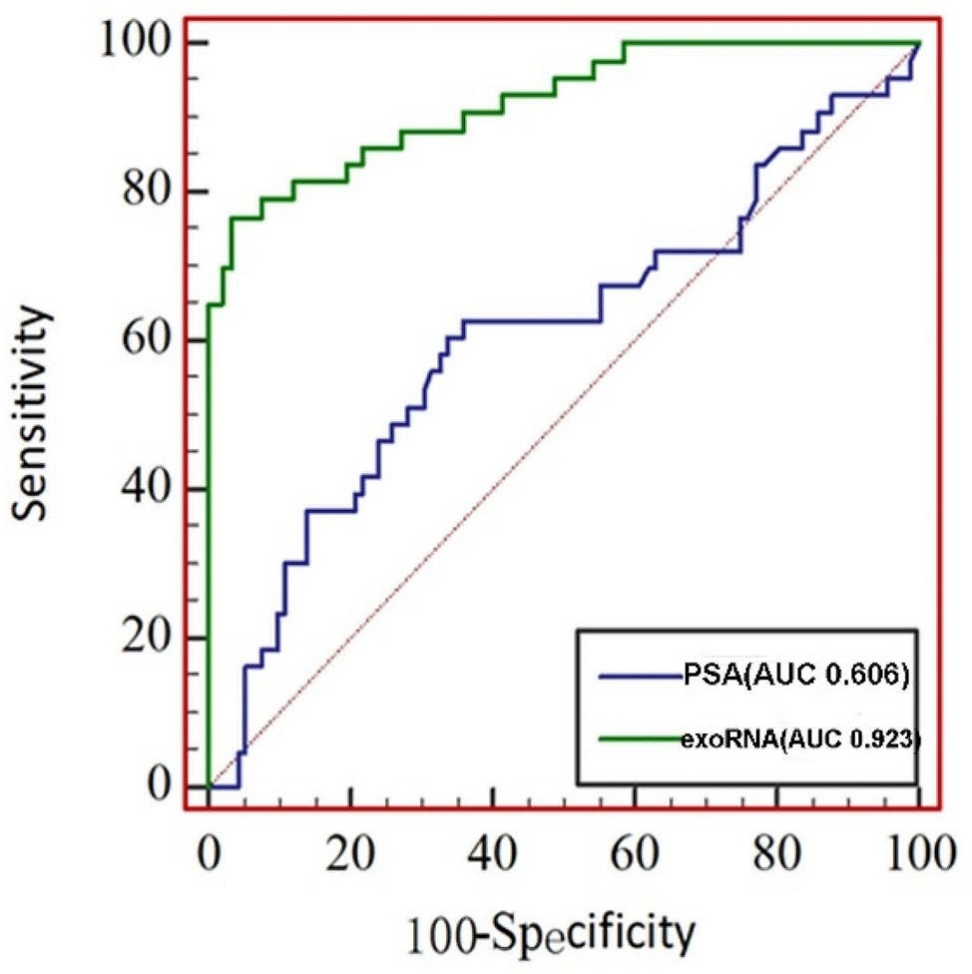
exoRNA model and PSA ROC curve

To enhance the diagnostic accuracy of prostate cancer (PCa) through the utilization of combined tumor markers, we conducted a multifactorial logistic regression subanalysis to establish a comprehensive diagnostic model based on urinary exosomal mRNAs(Table 1). We employed the logistic regression method in SPSS software to summarize and generalize the findings. The results of the multifactorial logistic regression analysis revealed that only PSA, *WWP1*, and *RAB5B* exhibited a statistically significant association with PCa (*P <* 0.05) (Supplemental Material 4). Based on this, we constructed a comprehensive diagnostic model using *WWP1* and *RAB5B*, utilizing the molecular exoRNAs derived from urinary exosomal mRNAs. When comparing the performance of this model with that of PSA alone (AUC = 0.606, sensitivity of 62.8%, specificity of 64.1%), we observed that the comprehensive diagnostic model using exoRNA achieved superior diagnostic efficacy. It yielded an AUC of 0.923 (95% CI: 0.878–0.968), with a sensitivity of 81.4% and a specificity of 89.1%. In conclusion, this newly demonstrated diagnostic model demonstrated a significant improvement in the diagnostic efficacy of prostate cancer (Table 2; Fig. 6).

**Table 2:** exoRNA model and PSA ROC curve data

Features	*N*	AUC	Sensitivity	Specificity	Jordon’s index	Optimal thresholds
exoRNA model (*WWP1* + *RAB5B*)	135	0.923	0.814	0.891	0.705	0.422
PSA	135	0.606	0.628	0.641	0.269	9.12

## Discussion

Prostate cancer is a prevalent type of cancer in men worldwide [1, 2]. However, current biomarkers used for early detection of prostate cancer, such as blood PSA levels and biopsy results, lack the necessary efficiency [3, 4]. Urinary exosomal mRNA, recognized for its easy collection and stability, offers a promising avenue as a potential source of biomarkers for prostate cancer. Our research explored this possibility by utilizing next-generation sequencing to analyze post-massage urinary exosomal mRNA, revealing distinct mRNA expression patterns between prostate cancer patients and individuals without the disease. In our investigation, we identified eight potential mRNA candidates that could distinguish between those with prostate cancer and those without.

Exosomes trap several biomolecules, including proteins and RNA, from the cytoplasm of the parent cell during their formation. They can both reflect pathological changes in the parental cells and mediate intercellular communication [36, 37]. Pisitkun et al. first identified exosomes in urine in 2004 [18]. S. Dijkstra’s study found that, after prostate massage urinary exosomes not only contained nucleic acid information about pathological changes in the prostate, but also the number of exosomes increased significantly [38]. To date, a variety of transcriptional substances in urinary exosomes have been found to play a role in prostate cancer and are potentially attractive in terms of prostate cancer biomarkers, e.g., *PCA3*, *MALAT1*, *HOTAIR*, *miR-1290*, and *miR-375* are some of the more common markers [10, 15]. In this study, we used ultracentrifugation to isolate urinary exosomes from urinary exosomes were isolated from post-DRE urine samples and examined the mRNA expression profiles. In addition, we found that mRNA expression differed between prostate cancer and non-cancer groups in the PSA range of 4–20 ng/ml, and constructed a comprehensive model of urinary exosomal mRNAs for the diagnosis of prostate cancer. Therefore, mRNAs of urinary exosomes are important candidate molecules for non-invasive biomarkers of prostate cancer.

mRNAs are relatively stable carriers of genetic information formed by selective splicing of DNA during its transfer from the nucleus to the cytoplasm, which can guide protein synthesis. Considering that the bilateral lipid membrane of urinary exosomes protects mRNA from degradation by RNA enzymes [39] and that urinary exosomes are easy to extract, mRNAs are well suited for clinical detection as a non-invasive biomarker. Woo et al. reported that *GATA2* mRNA in urinary exosomes could be used as a marker for predictive diagnosis of clinically significant prostate cancer [40]. To date, reports on urinary exosomal mRNA as a biomarker for prostate cancer are still scarce. In this study, urinary exosomes were taken from prostate cancer patients after massage, which can better reflect the tumor characteristics, and mRNAs with significant differences were screened by expression using next-generation sequencing, which is a mature and reliable technology. The TOP 10 mRNAs with the most significant differences in expression related to prostate cancer were taken for qRT-PCR and electrophoresis, and finally, 8 urinary exosomal mRNAs were identified, and the calculated diagnostic effect was superior to blood PSA. Blood PSA is recognized as an effective indicator of diagnostic markers for prostate cancer. However, in clinical practice, factors such as prostatitis, benign prostatic hyperplasia, acute urinary retention, and urethral manipulation lead to a positive result of PSA in the blood that does not necessarily imply the presence of prostate cancer. Batra et al. found that the diagnostic efficacy of PSA in the blood was not very high (sensitivity 70–90%, specificity 20–40%, and AUC 0.55–0.70) [41]. Our data suggest that a comprehensive model of urinary exosomal mRNAs (sensitivity 70–90%, specificity 20–40%, AUC 0.92), is better for the diagnosis of prostate cancer. However, due to the small sample size in this study, using urine exosome ribonucleic acid (mRNA) detection as an alternative to prostate biopsy in a clinical setting is currently not practical. Urinary exosome ribonucleic acid (mRNA) may have the potential to be a new area of research interest and could be a valuable early diagnostic marker for prostate cancer.

In this study, we constructed a comprehensive diagnostic model of 2 mRNAs (*WWP1*, *RAB5B*), which was significantly better than the PSA index in blood. The results of logistic regression analysis and ROC curves showed that these 8 mRNAs could also play an important role in diagnosis as independent risk factors for PCa. Among them, the AUC of *WWP1* was 0.906, the AUC of *RAB5B* was 0.880, and the AUC of *NRSN2* was 0.856, and all of them had high diagnostic efficiency. In addition, previous studies have shown that these eight mRNAs have important functions in a variety of tumors [27–34]. According to the results of this study, the constructed comprehensive model has diagnostic value for early prostate cancer. Urinary exosome-derived mRNAs are an important resource for studying non-invasive biomarkers of prostate cancer. However, further investigation is needed to examine the regulatory mechanisms of urinary exosomal mRNAs in prostate cancer.

Our study has some limitations. First, in practice, prostate cancer patients contain a wide range of staging, pathologic types, and different blood PSA ranges. It is unclear whether urinary exosome-derived mRNA can be used to diagnose prostate cancer of specific pathologic types and blood PSA over 20 ng/ml. Secondly, the sample size of this study is still small and needs to be further validated with the help of larger multicenter studies for better application in clinical practice. In addition, we did not conduct a follow-up study to determine the relationship between urinary exosome-derived mRNA and the progression of prostate cancer. In addition, the regulatory effects of diagnostic models on prostate cancer need to be validated by cellular and animal experiments, whereas the mechanisms by which urinary exosomal mRNAs regulate prostate cancer need to be further investigated. Finally, urinary exosomes may carry proteins and RNAs on their surface that have the potential to cause false-positive or negative results, and we did not use the appropriate enzymes for this. We expect that more studies will advance this field in the future and provide new strategies and methods for the early diagnosis and treatment of prostate cancer.

## Conclusions

The expression levels of urinary exosomal mRNAs following DRE exhibit a correlation with early-stage PCa and hold promise as a dependable biomarker for PCa. Our constructed diagnostic model utilizing urinary exosomal mRNAs surpasses the efficacy of the traditional blood PSA test, thereby offering a novel avenue for the advancement of early PCa biomarker research. Nevertheless, in terms of clinical applicability and diagnostic value, further prospective investigations are eagerly anticipated. These future explorations will not only advance the field but also provide new strategies and methodologies for the early detection and treatment of prostate cancer.

### Electronic supplementary material

Below is the link to the electronic supplementary material.

Supplementary Material 1

## Data Availability

The datasets generated and/or analyzed during the current study are available from the corresponding author (chengjiwen@stu.gxmu.edu.cn and wangfubo@gxmu.edu.cn) upon reasonable request.

## Abbreviations

PCa: Prostate cancer
lncRNAs: Long non-coding RNAs
DRE: Digital rectal examination
FBS: Fetal bovine serum
K-SFM: Keratinocyte-serum-free medium
TEM: Transmission electron microscopy
NTA: Nanoparticle-Tracking Analysis
PBS: Phosphate buffer solution
WB: Western Blot
qRT-PCR: Quantitative reverse transcription–polymerase chain reaction
PSA: Prostate-specific antigen
AUC: Area under the receiver operating characteristic curve
