# Supplementary material for: Unveiling potential: urinary exosomal mRNAs as non-invasive biomarkers for early prostate cancer diagnosis
Source: BMC Urol. 2024 Aug 1;24:163. doi: 10.1186/s12894-024-01540-6 (PMC11292860; doi:10.1186/s12894-024-01540-6)
Supplement: Supplementary file 1 — Supplementary Material 1 [file 12894_2024_1540_MOESM1_ESM.pdf]

Supplemental information

Unveiling the Potential: Urinary Exosomal mRNAs as Non-Invasive Biomarkers for Early Prostate Cancer Diagnosis

Jiayin Yu[a,1],Chifei Yu[b,1],Jiangkang Xian[c,1],Guanglin Yang[b,1], Shubo Yang[a],Shuting Tan[a],Tingting Li[a],Haiqi Liang[a],Qihuan He[a],Faye Wei[a],Yujian Li[a],Jiwen cheng[a,*],Fubo Wang[a,d,*]

a.Department of Urology, First Affiliated Hospital of Guangxi Medical University,22 Shuangyong Road, Nanning, Guangxi,530021, P.R.China.

b. Affiliated Tumor Hospital of Guangxi Medical University, Nanning, Guangxi, 530021, P.R.China

c.Department of Urology, The Second Affiliated Hospital of Fujian Medical University, Quanzhou, China

d.Center for Genomic and Personalized Medicine, Guangxi Key Laboratory for Genomic and Personalized Medicine, Guangxi Collaborative Innovation Center for Genomic and Personalized Medicine, Guangxi Medical University, Nanning, Guangxi, 530021, P.R.China.

[*] **Corresponding author.**

J.W Cheng, First-Corresponde, chengjiwen@stu.gxmu.edu.cn

F.B Wang, Co-Corresponde, wangfubo@gxmu.edu.cn

Jiayin Yua, Chifei Yu, Jiangkang Xianc, Guanglin Yang, these authors contributed equally to this work and should be considered co-first authors.

Supplemental Material 1

Visualized each exon of the genes using Integrative Genomics Viewer (IGV)

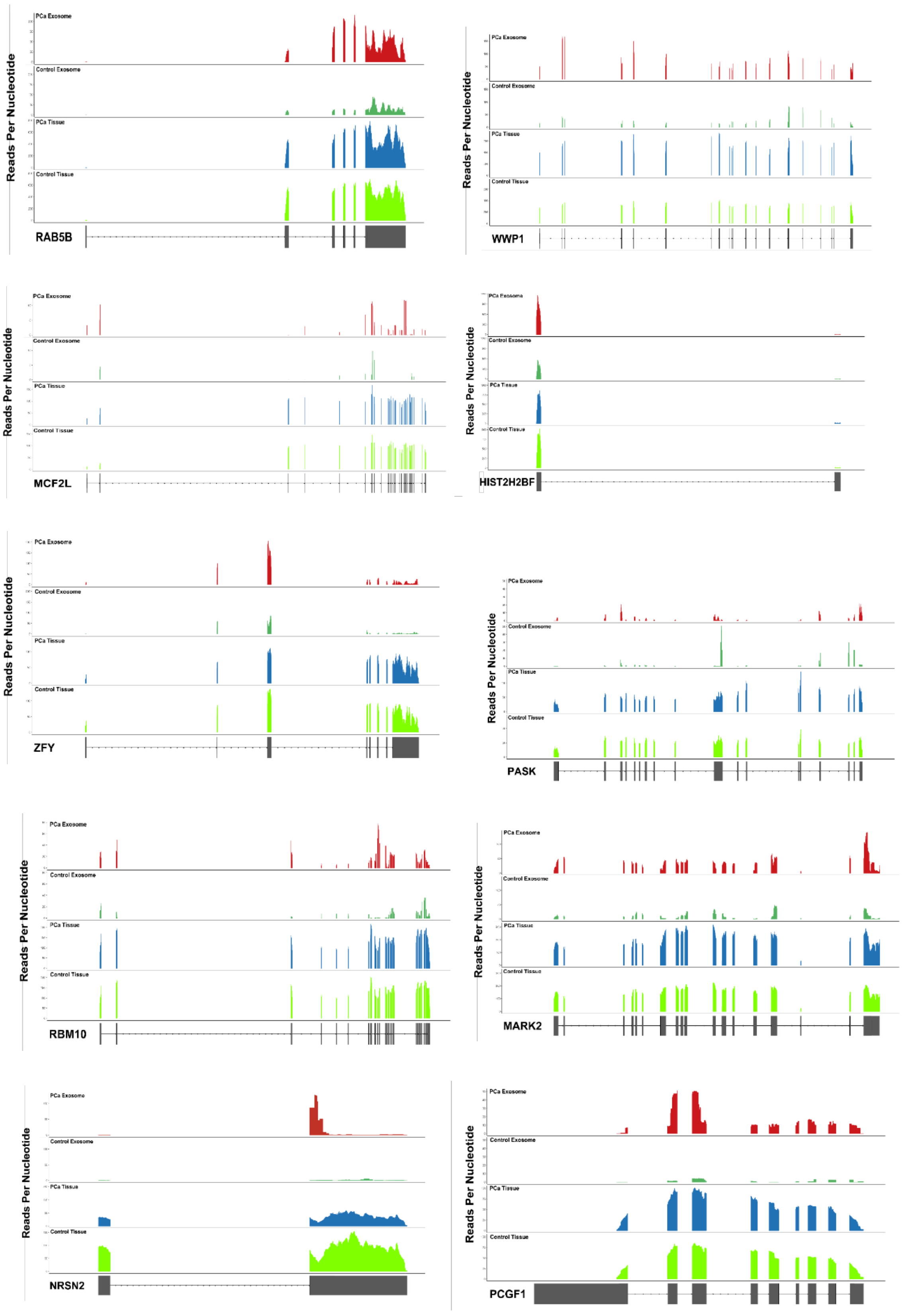

Supplemental Material 2

Specific primers and probes

Number	Primer Nam	Primer Sequence	Length	Probe Name	Probe Sequence	Fluorescent Group
1	rab5b-F2	GCCATGACTAGCAGAAGCACA	87	rab5b-P2	CTAGGCCCAATGGGCAACC	FAM
	rab5b-R2	GATTCTCCCAGCAGGACCAA				
2	rab5b-F3	TCCTCACCCAGTCCGTTTG	115			
	rab5b-R3	GCTTGGGCACCCCTGTAGTA				
3	wwp1-F7	TAATGCGTCTGTCACGGGTAC	107			
	wwp1-R7	AAGTCAGTATTTCTTGAACTGGAGG				
4	wwp1-F12	TGAAGAACCCCTGCCAGAA	104	wwp1-P12	TTATGATCAACAAAGTACCTTAC	FAM
	wwp1-R12	TGCGAGGATCTTTGAATGTTG				
5	mcf2l-F2	TGACTGTTGGAGATTTATCCTGTG	108			
	mcf2l-R2	ACATCAATTTGGTGATCCGTTT				
6	mcf2l-F13	TTACCTGCTGGCCTCACAAC	112	mcf2l-P13	ACAAGTGCCAGTCCCAGGAC	FAM
	mcf2l-R13	CTGGATCTTATTTTCCGCACC				
7	hist2h2bf-F2	CCCAGAGTTACCACAGGAAGACA	86	hist2h2bf-P2	CTCTTGACTCACAACAGCCACA	VIC
	hist2h2bf-R2	AAGTTGGACTCAGAGGGGACAG				
8	zfy-F3	TGACCCAGACTCAGTTGTAATCC	104			
	zfy-R3	CATTTTCAGATACATCTGCCTCTTC				
9	zfy-F4	TGGATGATGCTGGCAAAATAG	142	zfy-P4	TTCCACTGGAGTGACCATCGA	FAM
	zfy-R4	TCTTCTCCAGGGTCAGCTTTAA				
10	mark2-F7	CCTTATGCTGCCCCAGAACT	99	mark2-P7	AACTCCTAGGCTCCACACATC	FAM
	mark2-R7	TCCGCTGACCAGTGTATAGAGGA				
11	mark2-F15	GACCAGCAGAATTTGCCCTAC	96			
	mark2-R15	GAACTTGCTGAAGATGCTCCC				
12	pask-F2	TTAACAGCCTTTGAAGAGGACC	125			
	pask-R2	TGTCTGTGGGCTGAGGAAAA				
13	pask-F7	TGTTCTGCACCATCAGTGGC	94	pask-P7	TCATCACCCTCCTGCCGGAT	VIC
	pask-R7	CCGTAACCAAACAGTGTCAGC				
14	rbm10-F12	CTACTACCAACAGGATGAGGGCTAT	111			
	rbm10-R12	CCTTTGGTTCCAGTGATGCC				
15	rbm10-F16	TACCTGTACTGGGATGGGGAGA	134	rbm10-P16	ATGTTCCCGCCCTGGAGCAGT	VIC
	rbm10-R16	TGAGCTGTCTTGGTCTTGTGCT				
16	nrsn2-F1	ATGCCGAGCTGCAATCGTT	115			
	nrsn2-R1	CAGTGCCTGCACAGTCCTCATA				
17	nrsn2-F2	ACATCTCCTTCAACCCGTCC	112	nrsn2-P2	CACTCCTTCCTCTGCATGACCT	VIC
	nrsn2-R2	GAATCTGATCCAAAGCCCTCAT				
18	pcgf1-F1	CAAGACATTCTGGCAAAGACAA	94			
	pcgf1-R1	GGTTTAGCATCAAGCGGTGAC				
19	pcgf1-F4	TGACAATGAAGTTCTCCCTGATC	86	pcgf1-P4	CTGCATGACCTTGGGCAAACC	JOE
	pcgf1-R4	GTAAAAGCAAAGGGGATGGC				
20	actb-F6	TGCGTTACACCCTTTCTT	150	actb-P6	[illegible]AAAACCTAACTTGCGCAGAAAA	FAM
	actb-R6	CTGTCACCTTCACCGTTC				

Supplemental Material 3

DNA gel electrophoresis of PCR products of eight genes.

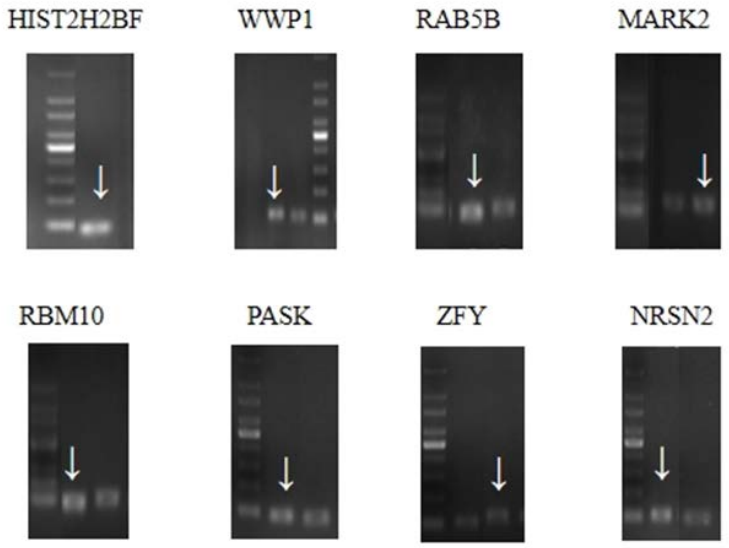

Supplemental Material 4

Multivariate logistic regression analysis results table.

Predictor	Estimate	SE	Z	p	Odds Ratio	Lower	Upper
(Intercept)	4.934	1.301	3.792	0.0	138.907	13.866	2414.107
RAB5B	-0.712	0.317	-2.244	0.025	0.491	0.251	0.884
HST2H2BF	0.031	0.225	0.138	0.89	1.031	0.655	1.6
PASK	0.107	0.243	0.442	0.659	1.113	0.691	1.82
MARK2	0.255	0.2	1.273	0.203	1.29	0.879	1.941
NRSN2	-0.326	0.269	-1.212	0.225	0.722	0.419	1.215
ZFY	-0.196	0.141	-1.392	0.164	0.822	0.616	1.08
RBM	0.005	0.228	0.021	0.983	1.005	0.644	1.594
WWP1	-0.619	0.188	-3.301	0.001	0.538	0.355	0.761

Supplemental Material 5

Table S1 mRNAs in urine exosomes and characteristics of patient samples.

Index	Overall sample	Puncture negative	Puncture positive	P 值
PSA				
Cases (%)	135(100)	92(68)	43(32)	0.047[#]
Median	8.83	8.24	10.26	
IQR	6.35-11.97	6.36-10.41	6.35-14.44	
WWP1				
Cases (%)	135(100)	92(68)	43(32)	＜0.001[#]
Median	40.06	20.615	212.28	
IQR	10.07-117.27	6.76-52.26	87.14-480.48	
RBM10				
Cases (%)	135(100)	92(68)	43(32)	＜0.001[#]
Median	14.78	9.89	57.5	
IQR	5.28-57.5	2.92-24.44	25.71-152.4	
ZFY				
Cases (%)	135(100)	92(68)	43(32)	＜0.001[#]
Median	23.58	13.81	75.18	
IQR	6.02-72.42	3.03-40.78	35.20-196.23	
NRSN2				
Cases (%)	135(100)	92(68)	43(32)	＜0.001[#]
Median	19.38	11.51	107.30	
IQR	6.58-95.66	4.10-30.74	51.17-263.32	
MARK2				
Cases (%)	135(100)	92(68)	43(32)	＜0.001[#]
Median	31.47	14.91	188.77	
IQR	9.42-188.77	6.77-64.68	48.10-521.58	
PASK				
Cases (%)	135(100)	92(68)	43(32)	＜0.001[#]
Median	11.54	7.05	48.14	
IQR	2.94-33.28	1.58-14.94	12.01-112.97	
HIST2H2BF				
Cases (%)	135(100)	92(68)	43(32)	＜0.001[#]
Median	88.75	61.1	409.01	
IQR	24.2-285.32	16.09-183.50	122.37-1432.91	
RAB5B				
Cases (%)	135(100)	92(68)	43(32)	＜0.001[#]
Median	62.64	32.41	211.28	
IQR	17.79-166.74	10.86-83.65	101.07-482.13	
